# Investigating Behavioral Responses to Mirrors and the Mark Test in Adult Male Zebra Finches and House Crows

**DOI:** 10.3389/fpsyg.2021.637850

**Published:** 2021-04-15

**Authors:** Pooja Parishar, Alok Nath Mohapatra, Soumya Iyengar

**Affiliations:** National Brain Research Centre, Gurugram, India

**Keywords:** mirror self-recognition, mark test, exploratory behavior, self-awareness, songbirds, zebra finches (*Taeniopygia guttata*), house crows

## Abstract

Earlier evidence suggests that besides humans, some species of mammals and birds demonstrate visual self-recognition, assessed by the controversial “mark” test. Whereas, there are high levels of inter-individual differences amongst a single species, some species such as macaques and pigeons which do not spontaneously demonstrate mirror self-recognition (MSR) can be trained to do so. We were surprised to discover that despite being widely used as a model system for avian research, the performance of zebra finches (*Taenopygia guttata*) on the mark test had not been studied earlier. Additionally, we studied the behavioral responses of another species of passerine songbirds (Indian house crows; *Corvus splendens*) to a mirror and the MSR mark test. Although a small number of adult male zebra finches appeared to display heightened responses toward the mark while observing their reflections, we could not rule out the possibility that these were a part of general grooming rather than specific to the mark. Furthermore, none of the house crows demonstrated mark-directed behavior or increased self-exploratory behaviors when facing mirrors. Our study suggests that self-directed behaviors need to be tested more rigorously in adult male zebra finches while facing their reflections and these findings need to be replicated in a larger population, given the high degree of variability in mirror-directed behaviors.

## Introduction

One of the most interesting aspects of cognition is self-awareness or to possess a sense of self. Despite the fact that self-awareness involves all senses including proprioception, self-generated vocalizations (Suarez and Gallup, 1987) and odors (Bekoff, 2001; Derégnaucourt and Bovet, 2016), visual self-recognition in mirrors using the “mark” test developed by Gordon Gallup for testing self-recognition in chimpanzees has been most widely used (Anderson and Gallup, 2015; Brecht and Nieder, 2020). For this test, an individual is marked with a contrasting dye or paint on a part of the body which can only be seen with the aid of a mirror. If the animal attempts to remove the mark while looking at the mirror, it is said to pass the mark test since it can recognize the image in the mirror as its own reflection. While humans pass the mark test easily, recent studies have suggested that this ability is not innate since mirror self-recognition develops approximately between 2 and 3 years of age across western and non-western countries. However, very few children of non-western rural areas (two out of 82 in Kenya) passed the test or displayed any orientation toward the mark, suggesting the role of cultural variations or differences in early exposure to mirrors (Broesch et al., 2011). Amongst the great apes tested, chimpanzees (Povinelli et al., 1993), orangutans (Suarez and Gallup, 1981), and bonobos (Anderson and Gallup, 2015) have been shown to demonstrate mirror-guided self-exploration of unseen parts of the body and to try and remove the mark while closely examining their reflections. However, very few (nine out of 92) chimpanzees passed the mark test. Whereas, most gorillas were shown not to pass the mark test, since they avert their gaze from reflections and do not explore themselves when faced with mirrors (Suarez and Gallup, 1981; Anderson and Gallup, 2015), those provided with an enriched environment were able to do so (Patterson and Cohn, 1994; Posada and Colell, 2007). Furthermore, macaques [*Macaca arctoides* and *Macaca mullata*; Gallup, 1970] did not pass the standard mark test, unless trained using an irritant laser (Chang et al., 2015) or by reinforcement training (Chang et al., 2017) to draw attention to the mark.

A variety of avian species, namely New Caledonian crows (Medina et al., 2011), Eurasian Magpies (Prior et al., 2008), zebra finches (Ryan, 1978; Iyengar et al., 2017), jungle crows (Kusayama et al., 2000), African gray parrots (Pepperberg et al., 1995), pigeons (Uchino and Watanabe, 2014), Java Sparrows (Watanabe, 2002), jackdaws (Soler et al., 2014), Clark's nutcrackers (Clary and Kelly, 2016), great tits (Kraft et al., 2017), keas and Goffin's cockatoos (Buuren et al., 2018), carrion crows (Vanhooland et al., 2019; Brecht et al., 2020), and Indian house crows (Iyengar et al., 2017; Buniyaadi et al., 2019) have been tested for their responses to mirrors. The general principle underlying these studies is that mirror self-recognition is an example of a higher cognitive function which has emerged across different species through convergent evolution (Reiss and Marino, 2001; Prior et al., 2008).

However, amongst these, only Eurasian magpies (Prior et al., 2008) and Indian house crows (Buniyaadi et al., 2019) were reported to demonstrate mirror self-recognition by passing the standard mark test. A different approach was used to test pigeons on MSR. Whereas, pigeons did not spontaneously pass the mark test (Epstein et al., 1981), they demonstrated mark-directed behavior after reinforcement learning. In these experiments, they were initially rewarded for pecking on a mark in the experimental chamber which was associated with a food reward. In subsequent sessions, the same mark was placed on their bodies and pecking on it was rewarded with food (Uchino and Watanabe, 2014). Although New Caledonian crows and African gray parrots did not demonstrate self-directed behaviors while observing their own reflections, they were able to retrieve food rewards using mirrors (Pepperberg et al., 1995; Medina et al., 2011). However, a recent study on pigeons (Ünver et al., 2017) has showed that the mirror-directed search behavior observed in both New Caledonian crows and African gray parrots (Pepperberg et al., 1995) should be interpreted cautiously since birds use peripheral vision rather than reflections for experiments on mirror-guided location of objects. Another member of the corvid family, that is, Clark's nutcrackers, are known to avoid caching their food in the presence of conspecifics (Clary and Kelly, 2011). Using this trait for studying self-recognition, nutcrackers were exposed to blurry or regular mirrors or a conspecific while birds were provided the opportunity to cache food (Clary and Kelly, 2016). Interestingly, nutcrackers only cached their food when they were alone or in the presence of the blurry mirror and not with conspecifics, suggesting that they may have been aware of their reflections. When the mark test was administered to the same set of birds, they demonstrated greater mark-directed preening when faced with mirrors, suggesting that they were able to recognize themselves in reflections. Even amongst magpies (Prior et al., 2008) and house crows (Buniyaadi et al., 2019) which were shown to pass the mark test, results were highly variable and only a small number actually passed the mark test. Furthermore, a recent study (Soler et al., 2020) tested magpies on mirror self-recognition using a protocol very similar to that used by Prior et al. (2008). They found that experimental birds paid more attention to their reflections compared to a non-reflective board and also performed self-directed and social behaviors more often in front of mirrors as shown in the earlier study. However, Soler et al. (2020) found that the majority of mark-directed behaviors were performed in front of the board rather than the mirror. Their findings suggest that there may be a high degree of variability on the performance of birds on the mark test. Alternatively, they have suggested that the magpies used tactile cues to detect the marks or that magpies in the earlier (Prior et al., 2008) study produced mark-directed behaviors by chance.

Amongst small passerines, the only species of birds to be tested on MSR with both paint marks and stickers are great tits (Kraft et al., 2017), which are known to produce other cognitively advanced behaviors such as a string-pulling task (Cauchard et al., 2013) and learning by observation (Brodin and Urhan, 2014). Although these birds spent more time observing their reflections, none of them passed the mark test. We decided to test zebra finches (*Taenopygia guttata*); another small passerine which despite having been used extensively in research for a variety of experiments, has only been tested once on visual self-awareness (Ryan, 1978). This study demonstrated that if given a choice, adult male zebra finches initially prefer to interact with conspecifics vs. their reflections but this preference is later transferred to their mirror images. Although zebra finches do not demonstrate tool use, they are capable of visually discriminating between dot and stripe patterns (Watanabe et al., 2008) and prefer blue colored nesting material over red or yellow (Muth et al., 2013). Furthermore, we were also interested in working on house crows, which are members of the Corvidae family, known for higher cognitive functions including possessing a theory of mind and tool use (Emery, 2004; Kenward et al., 2006). Both zebra finches and house crows are highly social birds which use their vocalizations to interact with conspecifics (Brown, 1985; Brainard and Doupe, 2002). However, at the time when these studies were undertaken, neither species had been tested on self-awareness using the mark test. Preliminary data from our lab had revealed that house crows appeared not to pass the mark test (Iyengar et al., 2017). Given the high degree of variability on the performance of corvids on the mark test (Prior et al., 2008; Soler et al., 2014; Buniyaadi et al., 2019; Vanhooland et al., 2019), we decided to perform a detailed examination of the performance on mirror self-recognition in house crows (*Corvus splendens*). Since zebra finches do not perform cognitively advanced tasks such as tool use, which has been observed in corvids such as New Caledonian crows (Kenward et al., 2006), we hypothesized that zebra finches would not pass the mark test. Preliminary data from our own initial experiments (Iyengar et al., 2017) and studies on other species of corvids [New Caledonian crows, jungle crows; (Kusayama et al., 2000; Medina et al., 2011)] suggested that house crows would also not pass the mark test or demonstrate MSR, despite the findings of Buniyaadi et al. (2019).

In earlier studies such as those on Asian elephants (Plotnik et al., 2006) and bottlenose dolphins (Reiss and Morrison, 2017), both of which passed the mirror mark test, baseline behavior was initially analyzed followed by observing the animals' responses to a mirror or a covered mirror. Once self-directed behaviors emerged, animals were tested to study whether they performed self-directed behaviors when they were marked with paint or sham-marked (touched on the area where the mark was to have been painted or not touched and exposed to the mirror or the covered mirror). For our studies, we modified the protocols used by Prior et al. (2008) and Soler et al. (2020) which were performed on magpies, neither of which recorded baseline behavior. In these studies, magpies were allowed to explore their mirror images (five sessions each, 30 min in length) or a similarly-sized non-reflective surface. This was followed by testing for mirror preference and then by the mark test for five sessions (each of 20 min duration), during which colored stickers were applied and birds' responses to their reflections or to the non-reflective surface were analyzed.

In our study, birds were provided eight sessions to habituate to the mirror (mirror exposure phase) wherein they were free to look behind but not physically go behind the mirror. During this period, we evaluated the progression of their performance on different social, aggressive/exploratory, self-directed, and contingency behaviors while facing their reflections. Following the mirror exposure phase, birds were exposed to four trials of test conditions after being marked or sham-marked (cf. Reiss and Marino, 2001) and their responses to a mirror or a non-reflective surface were tested. In most studies on MSR, animals or birds are only tested once or twice (Prior et al., 2008; Kraft et al., 2017; Buniyaadi et al., 2019; Soler et al., 2020). Testing the birds multiple times in the marked and sham-marked conditions enabled us to test whether mark-directed behavior was spontaneous or could be learnt over the trials. Furthermore, we chose two sites (on the head and neck) to paint or apply a sticker to exclude self-preening which had been performed by chance. Despite designing a rigorous protocol for assessing MSR in house crows and zebra finches, our results demonstrated that house crows do not recognize themselves in mirrors. Although a small number of zebra finches (two out of six) preened themselves near the mark while facing their reflections,- we were unable to conclude that they demonstrated MSR.

## Methods

A total of six adult male zebra finches (*Taenopygia guttata*) and five adult house crows (*Corvus splendens*) were used for the mirror self-recognition experiments. All experimental birds were housed in aviaries in the Animal Facility at the National Brain Research Centre, Manesar and experimental protocols were approved by the Institutional Animal Ethics committee in accordance with guidelines laid down by the Committee for the Purpose of Control and Supervision of Experiments on Animals (CPCSEA), India and compliant with international standards on animal welfare. All birds were identified by plastic leg bands and had not been used for any other experiments prior to testing for MSR. Whereas, zebra finches had been bred in our aviaries, the house crows were wild-caught and housed at the Animal Facility, NBRC for ~2 months prior to the MSR experiments. The aviary for zebra finches was maintained at a temperature of 25–29°C throughout the year and a 12L:12D light cycle. House crows were housed individually in cages in a separate aviary with natural lighting, maintained at 25–32°C. Most of the experiments to test MSR were performed between May and September, 2014 when the daylight hours are the longest in India and the light-dark cycle was between 13-16L:11-8D, to avoid behavioral changes elicited by alterations in the circadian rhythm.

### MSR Task for Zebra Finches

**Figure 1:**
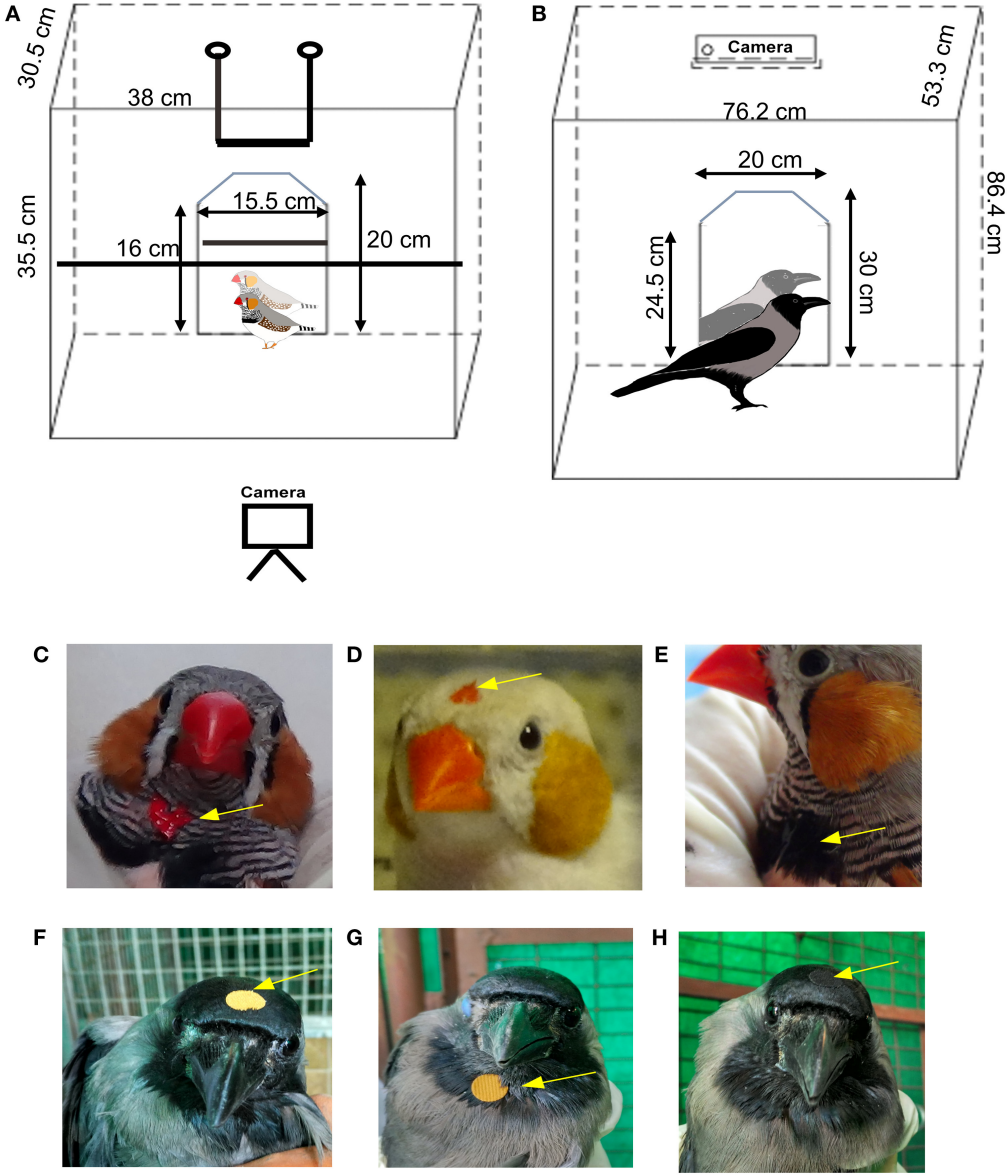
Schematic diagram of the behavioral setup used for testing mirror self-recognition in passerines and placement of the mark. **(A)** For zebra finches, two perches were placed in the cage and the camera was placed in front of the cage facing the mirror whereas for **(B)** house crows, the perch was removed during the MSR tests and the camera was placed above the cage. Placement of the “mark” on **(C–E)** zebra finches and **(F–H)** house crows. Arrows indicate the position of a red dot on a male zebra finch's **(C)** throat or **(D)** head. **(E)** A black dot was painted on the tuft of black feathers on the throat of zebra finch as a control to test whether they would attempt to remove the mark (BlThrMirr control, see text for details). A yellow sticker was placed on the **(F)** head or **(G)** throat area of a house crow for the MSR test. **(H)** The arrow indicates the position of a black sticker placed on the head as a control (BlHdMirr control condition) for house crows.

Six adult male zebra finches which had no prior exposure to reflective surfaces were housed in large cages, with unlimited access to food and water prior to the experiments and between trials. Birds were placed singly in a cage with a perch (Figure 1A) housed in a closed chamber which was visually and acoustically isolated from other birds. A mirror (20 cm by 15.5 cm) was introduced in the cage and their responses to their reflections were recorded for 30 min, using a video camera (Sony HDR-CX240EB). The method that we used was based on those followed by Prior et al. (2008) and Soler et al. (2020) wherein birds were first exposed to a mirror (mirror exposure phase) followed by a test phase consisting of interleaved trials of sham mirror/covered-mirror, covered-mirror mark and the mark test (Reiss and Morrison, 2017). During the mirror exposure phase, birds were exposed to and habituated to the mirror for the first 4 days of the week. For this phase, birds were placed in the testing chamber with the mirror for a total of eight sessions (two 30-min sessions per day) separated by a gap of 4–5 h every day for 4 days. Thus, experimental zebra finches were exposed to the mirror for a total of 4 h for habituation. If birds showed no signs of stress or aggression toward the mirror, they were subjected to the mark test. The mirror exposure phase was followed by a test phase, during which birds were marked with paint or sham-marked (see below). As was done for the mirror exposure phase, two trials were performed daily, separated by a gap of 4–5 h, and the experiment was repeated 5 days a week. The test phase comprised of five conditions each of which were tested four times, three with a mirror (Mirr) and two with a non-reflective board (Brd) placed in the cage as a control. Normal (gray) adult male zebra finches have alternating black and white striped feathers on their throats, with a tuft of black feathers at the midpoint. They were very sensitive to tactile stimulation, and removed small stickers placed on the feathers under their beak within seconds. Therefore, red or black odorless, non-toxic water-based tempera colors (washable paint, style: 54–1205, Crayola, Funskool) were used to paint spots of color on the throat or head for the mark test (Prior et al., 2008; Buniyaadi et al., 2019) (Figures 1C–E) which could not be observed by the bird unless in a reflection as given below:

1. Red spot on the throat facing a mirror: RedThrMirr
2. Red spot on the forehead facing a mirror: RedHdMirr
3. Black spot on the throat facing a mirror: BlThrMirr
4. Red spot on the throat facing a board: RedThrBrd
5. Feathers on the throat were stroked with a brush (sham): CtrBrd

For each condition, while one experimenter gently held the birds' heads and beaks in such a way that their eyes were covered, the other painted the mark on their throat or forehead (for the RedThrMirr, RedHdMirr, BlThrMirr, and RedThrBrd conditions) or stroked feathers on the throat [for the CtrBrd condition; Prior et al., 2008; Kraft et al., 2017)]. Whereas, the red spots were used to study whether zebra finches could recognize themselves in mirrors, we assumed that the black spot (painted on black feathers on the throat) served as a negative control for color. For two zebra finches which had white patches on their necks, white spots were painted on their necks as controls for color. In the last condition, birds were sham-marked by stroking feathers on the throat with a Q tip (also used to apply the paint) to study whether touch rather than visual stimuli evoked preening on the throat. After painting different colored spots for each condition, zebra finches were placed singly in the test cage in a sound-proof isolation chamber facing the mirror or the non-reflective board and the video-recording was started. Trials of all experiments were interleaved randomly and counterbalanced so that the zebra finches did not get habituated to any of the experimental conditions.

### MSR Task for House Crows

House crows (*n* = 5; three males and two females) were housed in cages 30″ × 21″ × 34″ in dimension with *ad libitum* access to food [bread, canned dogfood (Pedigree), boiled egg, multivitamins] and water. Prior to the experiments on self-recognition, they were placed singly in a cage for 2 days for habituation, during which time they were also provided food and water (Figure 1B). Following habituation, food, water and perches were removed from the cage during behavioral recordings performed during mirror exposure and test phases, as described above for zebra finches. A screen was placed between the house crow being tested and other crows in the aviary. Whereas, experimental birds were visually isolated from other house crows, they were able to hear them vocalize during experiments. House crows have black plumage on their wings, head and just under the beak and gray feathers on their bodies and the back of the head. Despite the fact stickers have been reported to provide tactile stimulation in jackdaws (Soler et al., 2014), we found that it took longer to paint spots of color on house crows and remove them after the experiments, leading to greater degrees of stress amongst these birds. Therefore, to study whether they recognize themselves in a mirror image, we placed yellow stickers (diameter, 9 mm; weight, 12–13 mg) on their foreheads or under the beak, which would be easily visible against their black plumage. For controls, similarly-sized black stickers were placed on their foreheads, which blended with the color of their feathers (Figures 1F–H). Care was taken to ensure that the house crows were unable to see where the mark was placed on their bodies, in the same manner as described for zebra finches by two experimenters. Additionally, stickers were placed on their wings and bellies to test whether they were capable of removing the “marks” if viewed directly, that is, by perceiving stickers as foreign objects. The mirror exposure period was followed by the test phase comprising of a set of three different conditions (four trials, 30 min each) during which crows were placed in front of a mirror or board (control), as follows:

1. Yellow sticker on the forehead with mirror: YelHdMirr
2. Yellow sticker on the throat with mirror: YelThrMirr
3. Black sticker on the forehead with mirror: BlHdMirr
4. Yellow sticker on the head with a non-reflective board: YelHdBrd
5. Feathers on the forehead were touched as if pasting a sticker facing a non-reflective board (sham): CtrBrd.

For all conditions, behavior was recorded with a Micromax handset (Canvas A110) 8-megapixel camera at 30 fps. Trials of all experiments were randomized and counterbalanced so that crows did not get habituated to any of the experimental conditions.

### Behavioral Analysis

The first 30 s of the video recordings were discarded to avoid measuring behaviors which may have been affected by the experimenters exiting the area near the experimental chamber or cages. A number of behaviors were scored, including (1) Social responses, which included the (i) time spent in front of the mirror (in seconds) and (ii) the number of songs and calls; (2) contingency testing, that is, behaviors performed in front of the mirror which enables the individual to perceive a visual—kinesthetic match between the behavior and its reflection (Morrison and Reiss, 2018). This includes the (i) number of head turns in front of the mirror, including those to one side and back to the midline in front (Supplementary Figure 1A) and from side to side. Head turns are used to explore the surroundings since birds' eyes are placed laterally (as described in the schematic; (3) mark-directed behavior including the number of times birds (i) preened their neck, (ii) scratched their neck or head using their claws, and (iii) preened their wings while facing the mirror (directed preening). We also analyzed the (4) preening behavior including the number of times birds (i) preened their neck, (ii) scratched their neck or head using their claws, and (iii) preened their wings while facing away from the mirror (undirected preening). Additionally, we analyzed (5) aggressive/exploratory responses by counting the number of times birds pecked on the mirror or the frame and (6) search responses wherein the (i) number of times birds looked behind the mirror and the (ii) number of times birds turned clockwise and anti-clockwise were counted.

For the statistical analysis of preening behavior, we combined preening using the beak and scratching/preening using claws. Furthermore, for comparison across different conditions in the test phase, in case of CtrBrd and RedThrBrd conditions, directed and undirected preening were collated since birds could not observe their reflections and randomly preened themselves in different parts of the cage. These were then compared with directed preening behavior in the mirror trials.

### Statistics

The One-Way Repeated Measures Analysis of Variance (RM-ANOVA) test was used to compare different conditions and test for statistical significance, followed by the Tukey's *post-hoc* test using SigmaPlot 14.0 (Systat Software, San Jose, CA), which gives adjusted *P*-values for pairwise multiple comparisons. Data obtained for preening (the most important self-directed behavior for demonstrating whether birds passed the mark test) was tested using the Fisher's exact test or by multiple comparisons using the Fisher's exact test with the Bonferroni correction using the R software [version 3.6.0; R library; RVAideMemoire (version 0.9-73. To test changes in the progression of behaviors such as time spent, vocalization, search responses, pecking, and head turns in the mirror exposure phase, data was fitted in a linear regression model to calculate the slope parameter, followed by a *t*-test for the trend analysis. A Welch's *t*-test was performed to compare data of contingency testing from mirror vs. board conditions for Y45 and G123, the two zebra finches in which preening increased near the marked area. The entire dataset that we obtained for these experiments was analyzed by one of the observers (ANM) who had not performed the experiments. Another observer analyzed 30 trials and the correspondence between their ratings (r value or inter-rater reliability using the Pearson product moment correlation test; SigmaPlot 14.0) was highly correlated [r range 0.979–0.996, n range 30–140, P range 10^−123^-10^−24^; (Clary and Kelly, 2016)]. Wherever there were doubts or disagreements regarding the data, the observers discussed the results to reach a consensus for the final analysis.

## Results

### Adult Male Zebra Finches

#### Mirror Exposure Phase

**Figure 2:**
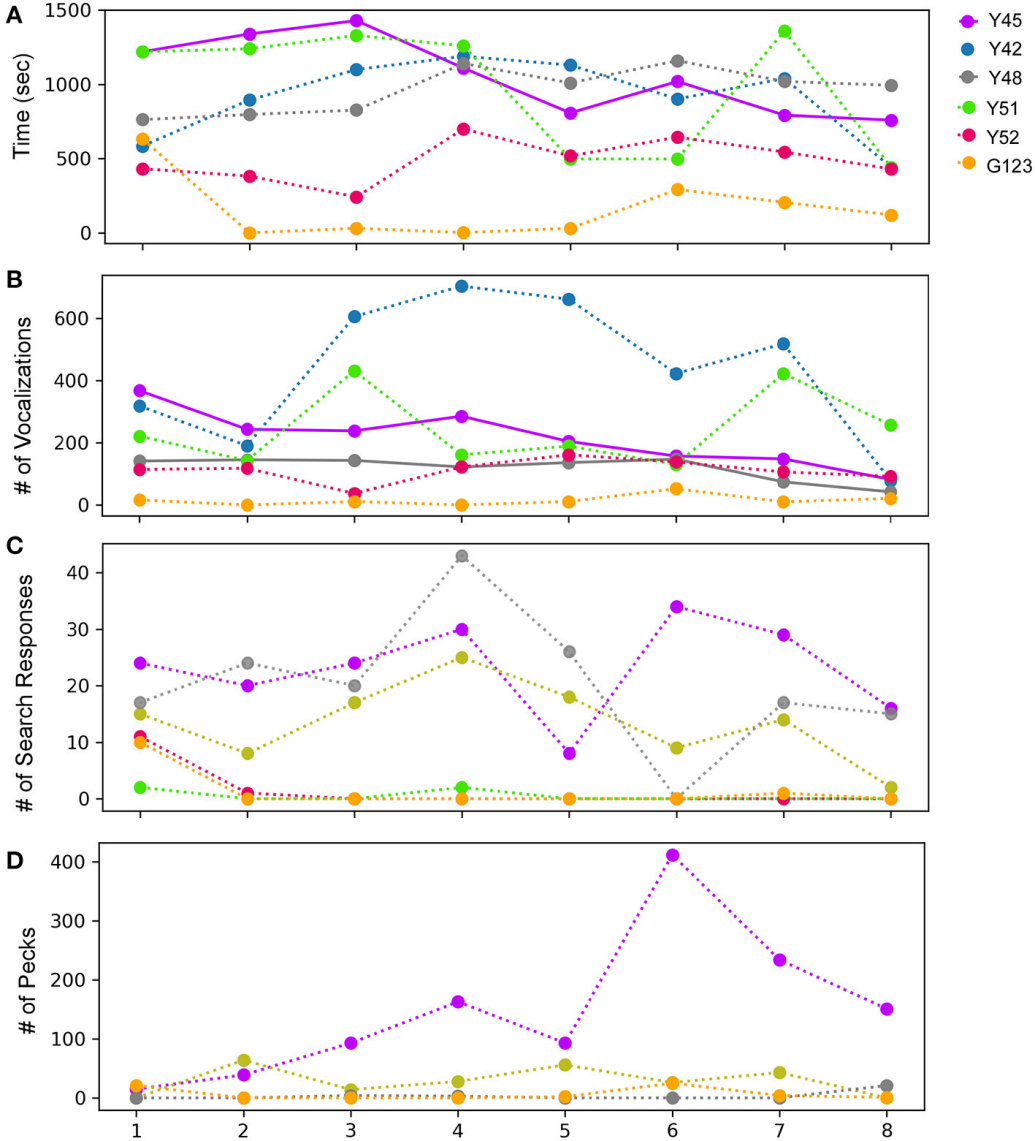
Behavior following observations of mirror images for adult male zebra finches across eight successive sessions during the mirror exposure phase. **(A)** Time spent while facing the mirror, **(B)** number of vocalizations (calls or songs) directed toward the mirror image, **(C)** number of search responses (looking behind the mirror and turning clockwise or anticlockwise) performed while facing the mirror, and **(D)** aggressive behavior, measured by the number of pecks on the mirror or on its sides. Colors indicate different birds, bold lines indicate significantly increasing or decreasing linear trends (*P* < 0.05; paired *t*-test) as trials progressed, which were highly variable across birds, whereas fainter dotted lines indicate no significant differences for each behavior.

During the mirror exposure phase, zebra finches were exposed to an uncovered mirror and their behavior was analyzed as described in the Methods section. Of the six birds tested, only one (Y45) demonstrated a significant decrease in the time spent in front of the mirror as the mirror exposure trials progressed (Figure 2A). Other birds used for this experiment also appeared to display a keen interest in their mirror images but did not show any trend as the trials progressed. The only exception to these results was one bird (G123), which spent the least amount of time facing the mirror compared to other birds.

Most of the birds produced vocalizations (calls and songs) directed toward their reflections during the initial trials. Interestingly, there was a significant decrease in vocalization by two of the birds Y45 (*t*-test, *t* = −5.723, *P* = 0.001) and Y48 (*t*-test, *t* = −2.862, *P* = 0.028; Figure 2B; Supplementary Video 1). These findings suggest that these birds adapt to their reflections over the course of mirror exposure. However, this behavior was variable in other birds and did not show any trend.

An analysis of search responses and aggressive behavior (pecking) during the mirror exposure phase did not demonstrate any significant changes in their progression in any of the birds. In three birds (Y45, Y48, and Y42) out of the six tested, the number of search responses in the initial trials were high, followed by a non-significant decrease in the last few trials (Figure 2C). The three other birds used for these experiments (Y51, Y52, and G123) displayed almost negligible search responses throughout the mirror exposure phase. We also did not find any significant trend in the progression of aggressive/exploratory behavior in any of the zebra finches used in our study. There was an increase in pecking in case of one of the birds (Y45) in the first six trials of the mirror exposure phase, followed by a decrease in the last two trials (Figure 2D).

**Figure 3:**
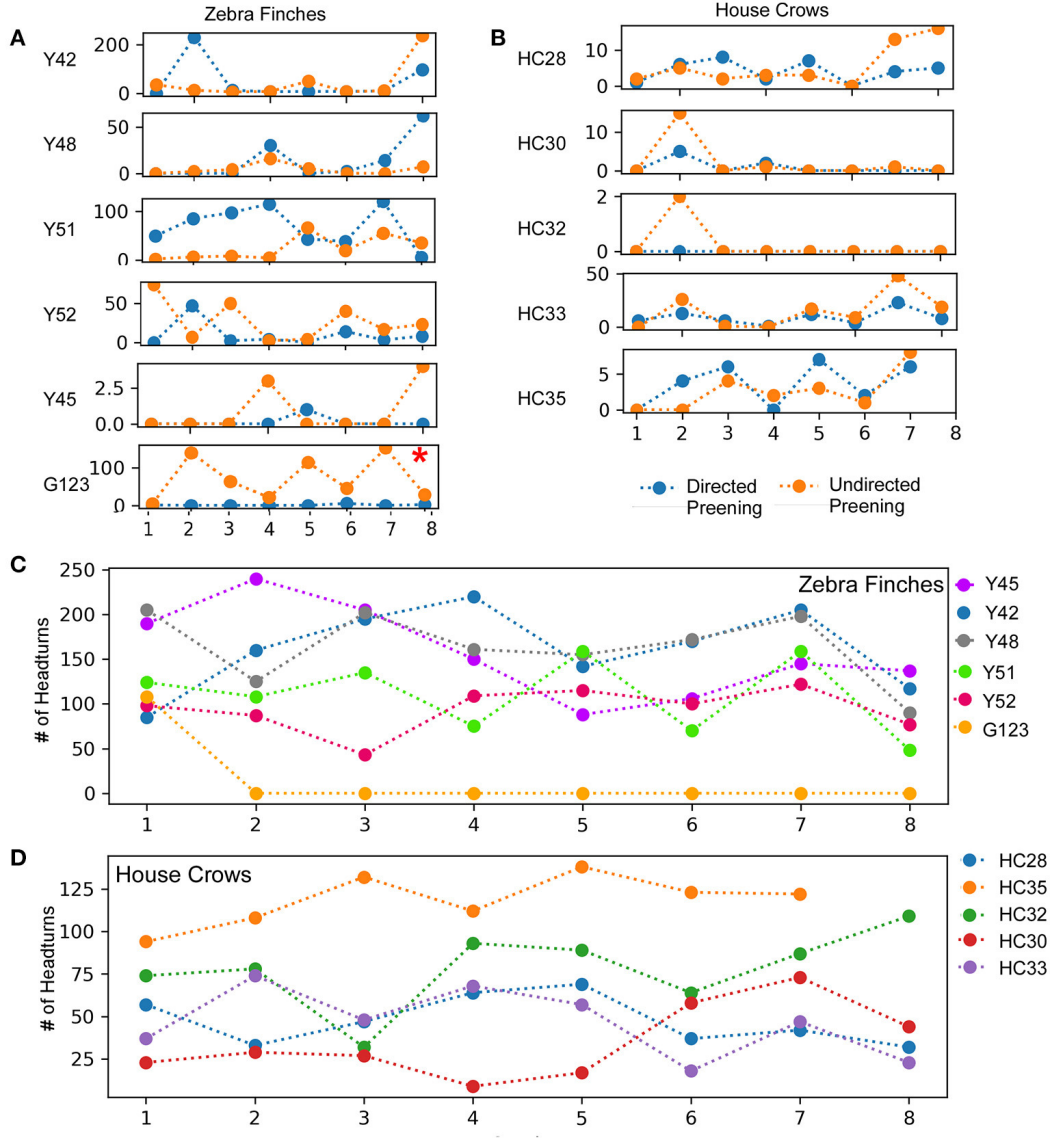
A comparison of directed and undirected preening across successive mirror exposure trials for **(A)** zebra finches and **(B)** house crows. Blue dots and lines indicate directed preening whereas orange dots and lines indicate undirected preening (*t*-test *, *P* < 0.05). The number of head turns in **(C)** zebra finches and **(D)** house crows during the mirror exposure phase did not change significantly across trials.

In birds, auto- or self-preening is an essential component of self-directed behavior. For zebra finches, this behavior is important for grooming and cleaning their feathers and is performed very frequently. To evaluate this behavior during the mirror exposure phase, we scored the number of times birds preened their head, neck or wings using their beak or claws while facing the mirror (directed preening) and also while facing away from it (undirected preening) and found that these behaviors were highly variable. In the mirror exposure trials, we found significantly high levels of undirected preening only in one bird (G123). Amongst other birds, Y48 demonstrated increased levels of directed preening in the last two trials. Whereas, Y51 also demonstrated increased levels of directed preening, this was only observed in the initial four trials and Y45 demonstrated negligible preening behavior (Figure 3A).

Most of the birds in our study demonstrated a large number of head turns, a contingency behavior, while facing the mirror during the mirror exposure phase. Although there were no significant differences in this behavior across trials, there was a decrease in the number of head turns in one zebra finch (Y45; *t*-test, *t* = −2.325, *P* = 0.059). Another zebra finch (G123) turned its head to view its reflection only in the first trial during mirror exposure, after which it did not perform this behavior even while facing the mirror. Taken together, by the end of the mirror exposure, all zebra finches performed fewer head turns than the number in the first trial (Figure 3C).

#### Comparison Between the Mirror Exposure and Test Phases

##### Attention Toward Reflections, Vocalization, and Contingency Testing

**Figure 4:**
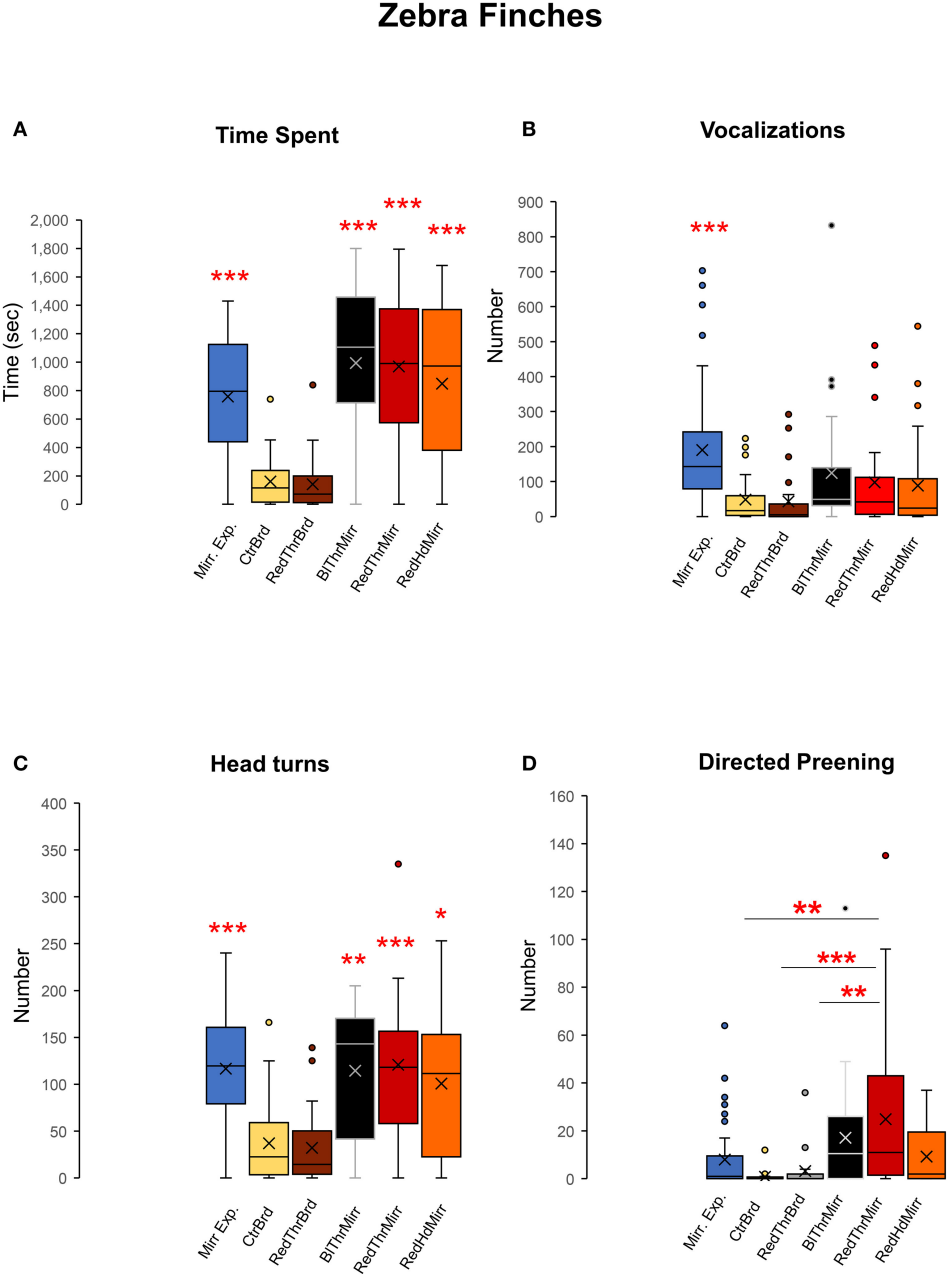
Attention paid to the reflection, social responses, contingency, and preening behaviors of zebra finches during the mark test. **(A)** Zebra finches (*n* = 6) spent significantly higher amounts of time facing the mirror during both mirror exposure and mirror test trials compared to facing a board of similar dimensions (control), suggesting that there was an increase in attention paid toward their reflections. **(B)** Zebra finches vocalized (sang and called) more toward their reflections during the mirror exposure period compared with other test conditions (*post-hoc* Tukey test ***, *P* < 0.001). **(C)** There was a significant increase in head turning (a contingency behavior) by all zebra finches used in our study while facing the mirror (*post-hoc* Tukey test *; *P* < 0.05, **; *P* < 0.01, ***; *P* < 0.001). **(D)** There was a significant increase in directed preening on the neck in the RedThrMirr condition compared to that in the CtrBrd, RedThrBrd, and mirror exposure period (*post-hoc* Tukey test; *P* < 0.01; **; *P* < 0.001***).

We exposed adult male zebra finches (*n* = 6) to their reflections in a mirror to record their reactions and evaluated their response to the mark test. All birds displayed a keen interest in their reflections by spending more time facing the mirror (inclusive of mirror exposure and test trials) compared to board trials One-way RM ANOVA, [F_(5,47)_ = 29.868], *P* < 0.001; Tukey's *post-hoc* test, *P* < 0.001; Figure 4A. The number of vocalizations directed toward birds' reflections was also found to be higher in mirror exposure trials compared to test trials One-way RM ANOVA, [F_(5,47)_ = 13.651], *P* < 0.001; Tukey's *post-hoc* test, *P* < 0.001; Figure 4B; Supplementary Video 1. Although the number of vocalizations (songs and calls) was also higher in mirror trials in the test phase (BlThrMirr; RedThrMirr and RedHdMirr; Figure 4B) compared to the board trials, these differences were not significant.

All zebra finches demonstrated a significant increase in contingency testing when faced with their reflections, compared to control conditions, while facing a board {Supplementary Video 2; Figure 4C; One-way RM ANOVA, [F_(5,47)_ = 19.716], *P* < 0.001; Tukey's *post-hoc* test}. This was measured by counting the number of head turns during each of the control and experimental conditions used in our experiment.

##### Exploratory/Aggressive and Search Responses

**Figure 5:**
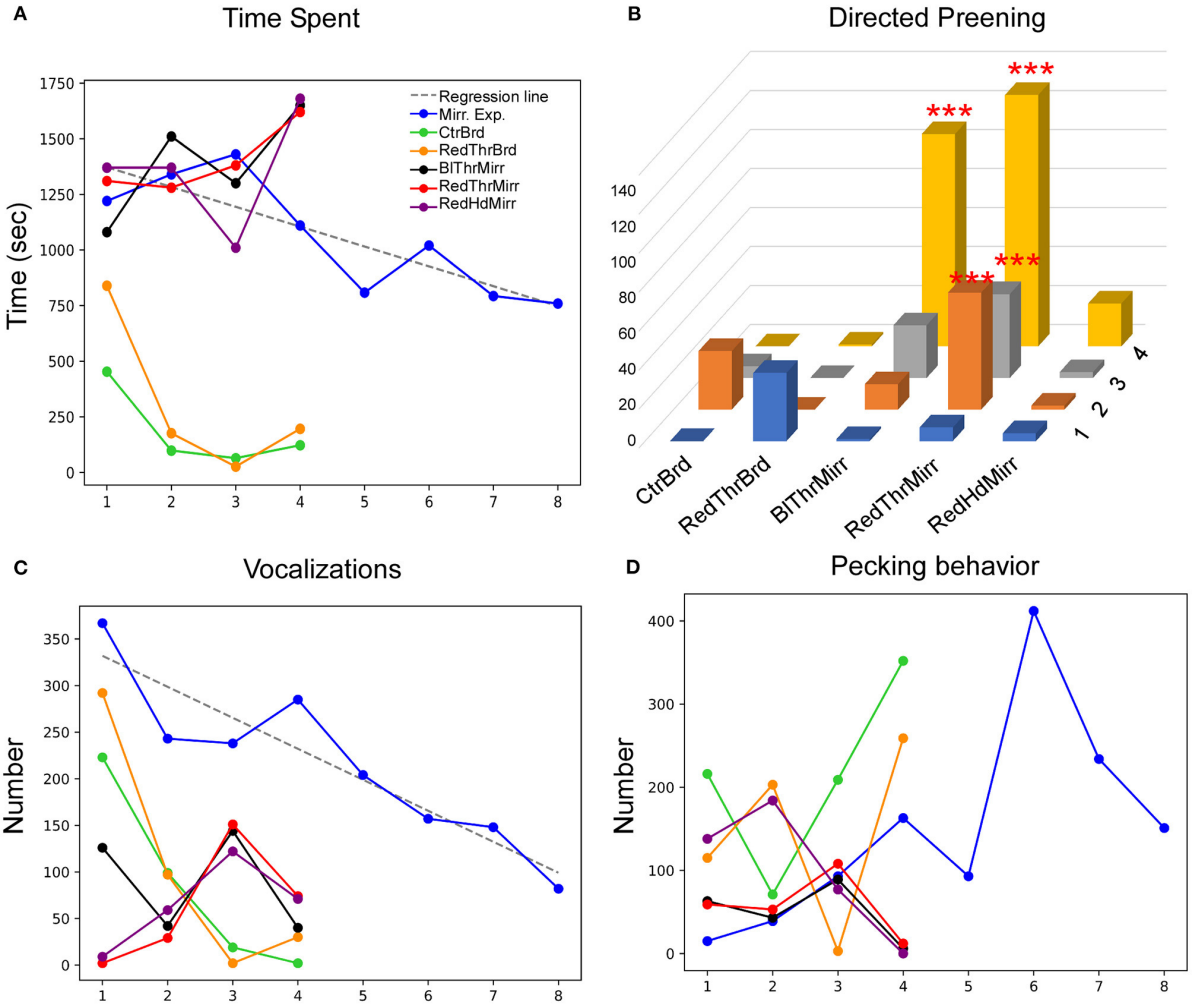
Behavioral response of an adult male zebra finch (Y45) during the MSR test. **(A)** This bird displayed a significant decrease in the time spent facing the mirror during mirror exposure trials. Additionally, this parameter decreased in conditions when the mirror was replaced with a board of similar dimensions. **(B)** There was a significant increase in preening on a red mark placed on the neck compared to controls. Furthermore, there was a significant increase in preening in Trial 4 when a black mark was placed on the neck (***; *P* < 0.001; Fisher's exact test). **(C)** There was a significant decrease in vocalization across different trials during the mirror exposure period (indicated by the dotted regression line). Vocalization also decreased in all other conditions (whether in the mirror or board conditions) by Trial 4. **(D)** There was an increase followed by a decrease in pecking on the mirror during the mirror exposure trials and this trend was seen in all test mirror conditions. However, the reverse trend was observed for the board conditions.

Unlike other species of birds such as magpies (Prior et al., 2008) and New Caledonian crows (Medina et al., 2011) which flapped their wings or fluffed their feathers to show dominance while viewing their mirror images, none of the zebra finches that we used in our experiments displayed such behaviors. However, four of the birds (Table 1) exhibited exploratory/aggressive behaviors toward the mirror or the board by pecking at the surface or on the frame. One bird (Y45) displayed unusually high levels of pecking in board trials (not significant; ns) indicating heightened interest toward the board, which was perhaps exploratory, rather than aggressive (Table 1; also see Figure 5D).

**Table 1:** Various behaviors performed by male zebra finches during mirror self-recognition experiments.

**Behavior**	**Bird**	**Mirror exposure**	**CtrBrd**	**RedThrBrd**	**RedThrMirr**	**BlThrMirr**	**RedHdMirr**
Vocalization	Y42	3495	556	512	1839	1269	1499
	Y45	1724	343	421	352	256	261
	Y48	950	124	30	339	226	124
	Y51	1953	19	1	21	425	15
	Y52	888	79	53	351	82	100
	G123	121	35	20	108	64	120
Head turns	Y42	1294	135	210	505	365	437
	Y45	1261	316	270	449	529	503
	Y48	1308	186	79	626	690	650
	Y51	878	105	152	385	600	304
	Y52	751	145	56	436	313	266
	G123	108	0	0	499	246	253
Exploratory/Aggressive	Y42	231	0	9	8	67	117
	Y45	1200	848	580	232	201	399
	Y48	28	119	97	88	112	80
	Y51	0	0	0	0	0	0
	Y52	0	0	0	0	0	0
	G123	53	0	0	3	4	24
Search responses	Y42	120	6	17	20	42	50
	Y45	185	27	24	60	52	60
	Y48	162	22	18	30	25	28
	Y51	4	3	0	0	0	0
	Y52	12	0	2	0	2	0
	G123	11	0	0	3	3	8
Directed preening	Y42	124	1	32	54	0	4
	Y45	0	0	37	259	164	33
	Y48	45	3	0	68	56	72
	Y51	150	3	0	31	75	73
	Y52	59	13	8	61	61	29
	G123	8	0	0	125	54	12
Undirected preening	Y42	263	26	134	125	30	6
	Y45	1	36	1	0	49	5
	Y48	8	33	90	46	14	32
	Y51	77	65	82	18	5	15
	Y52	108	51	231	229	132	172
	G123	343	28	14	9	9	9

Apart from exploratory/aggressive behavior, birds (*n* = 3; Y42, Y45, and G123) also displayed other search behaviors such as looking behind the mirror and turning clockwise and anti-clockwise (Table 1) which decreased in the test trials (ns).

##### Self-exploration

Zebra finches mostly used their beaks to preen themselves rather than scratching/preening themselves using their claws. We analyzed self-exploration in zebra finches by counting the number of times they preened themselves on their head and neck. There was a significant increase in directed preening in trials for the RedThrMirr condition wherein birds could view themselves in the mirror and a red spot had been painted on their throat {One-Way RM ANOVA; [F_(5,47)_ = 6.918], *P* < 0.001; Tukey's *post-hoc* test *P* < 0.01; Figure 4D}, whereas there were no differences in undirected preening across different conditions (Table 1). These results stemmed from significant increases in directed preening in the RedThrMirr condition vs. the board conditions (CtrBrd, RedThrBrd) and mirror exposure trials. Most of the zebra finches (*n* = 5) exhibited high levels of preening in the mirror exposure trials except for Y45. Amongst the tested birds, only Y45 and G123 demonstrated preening near the mark in mirror test trials (discussed below). We did not find any difference in the preening behavior of other birds (Y42, Y48, Y51, and Y52) in the board test trials (Directed and Undirected Preening; Table 1) and mirror test trials, suggesting that this behavior may be in response to somatosensory stimulation caused by touching feathers on the neck. We also found that directed preening was lower for the RedHdMirr compared to the RedThrMirr condition in most of the birds (*n* = 5).

### Mark Test

#### Analysis of the Behavior of the Two Zebra Finches Which Appeared to Display Mark-Directed Behavior

##### Y45

This bird clearly demonstrated a high degree of interest in its mirror image in all test trials, demonstrated by significantly more time spent in all mirror conditions (mirror exposure and test) compared to the board conditions (Tukey's *post-hoc* test, *P* < 0.05). Interestingly, it also displayed adaptation to the mirror image as the mirror exposure trials progressed (*t*-test, *t* = −3.839, *P* = 0.008; Figure 5A), since the amount of time spent in front of the mirror decreased in the last few mirror exposure trials.

Whereas Y45 displayed negligible preening in mirror exposure trials (Table 1), this parameter increased in mirror test trials (Figure 5B). We observed increased mark-directed preening only in the RedThrMirr condition (Fisher's exact test, *P* < 0.0001; Supplementary Figure 1B) compared to the mirror exposure and control condition. Preening was very low in the initial trials of both BlThrMirr and RedThrMirr conditions but increased in the last three trials. Multiple comparisons of different test conditions vs. trials demonstrated that mark-directed preening was significantly higher in the last three sessions of the RedThrMirr than in the RedThrBrd condition (pairwise comparison using Fisher's exact test, *P* < 0.0001) and in the last two trials of the RedThrMirr condition compared with the CtrBrd condition (pairwise comparison using Fisher's exact test, *P* < 0.05; Supplementary Video 3). Although we assumed that a spot of black paint on feathers under the neck (the BlThrMirr condition) could be used as a control for color, Y45 displayed high levels of mark-directed behavior in this condition as well. Significantly high mark-directed preening was also observed in the last trial of the BlThrMirr condition compared to the RedThrBrd condition (pairwise comparison using Fisher's exact test, *P* < 0.0001) and the last two trials compared to the CtrBrd condition (pairwise comparison using Fisher's exact test, *P* < 0.0001).

There was a steep decline (*t*-test, t = −5.723, *P* = 0.001; Figure 5C) in the number of vocalizations directed toward the mirror image as the mirror exposure trials progressed, implying that Y45 may have perceived that the reflected image was not a conspecific bird or may have adapted to the “conspecific's” presence by the end of the mirror exposure trials. Overall, vocalization was lower in all test trials compared to the mirror exposure trials {One-Way RM-ANOVA, [F_(5,27)_ = 4.465], *P* = 0.011; Holm-Sidak *post-hoc* test; *P* < 0.05 for all test trials except for RedThrBrd}. Vocalization was higher in the initial trials for both board conditions (CtrBrd and RedThrBrd, Figure 5C), followed by a decrease from the next trial onward, which may have occurred as a result of the bird anticipating the presence of the mirror and its mirror image when placed in the experimental set-up. Additionally, it displayed significantly high mirror-directed contingency behavior (Welch's *t*-test, *t* = 2.410, *P* = 0.0320; Table 1) compared to that in the board condition suggesting that it was trying to match visual and kinesthetic input.

Lastly, Y45 also demonstrated mirror-image directed pecking (Figure 5D; including pecking on the mirror and on its frame). However, there was no significant change in the levels of pecking during the mirror exposure trials as well as in test trials across all conditions.

##### G123

**Figure 6:**
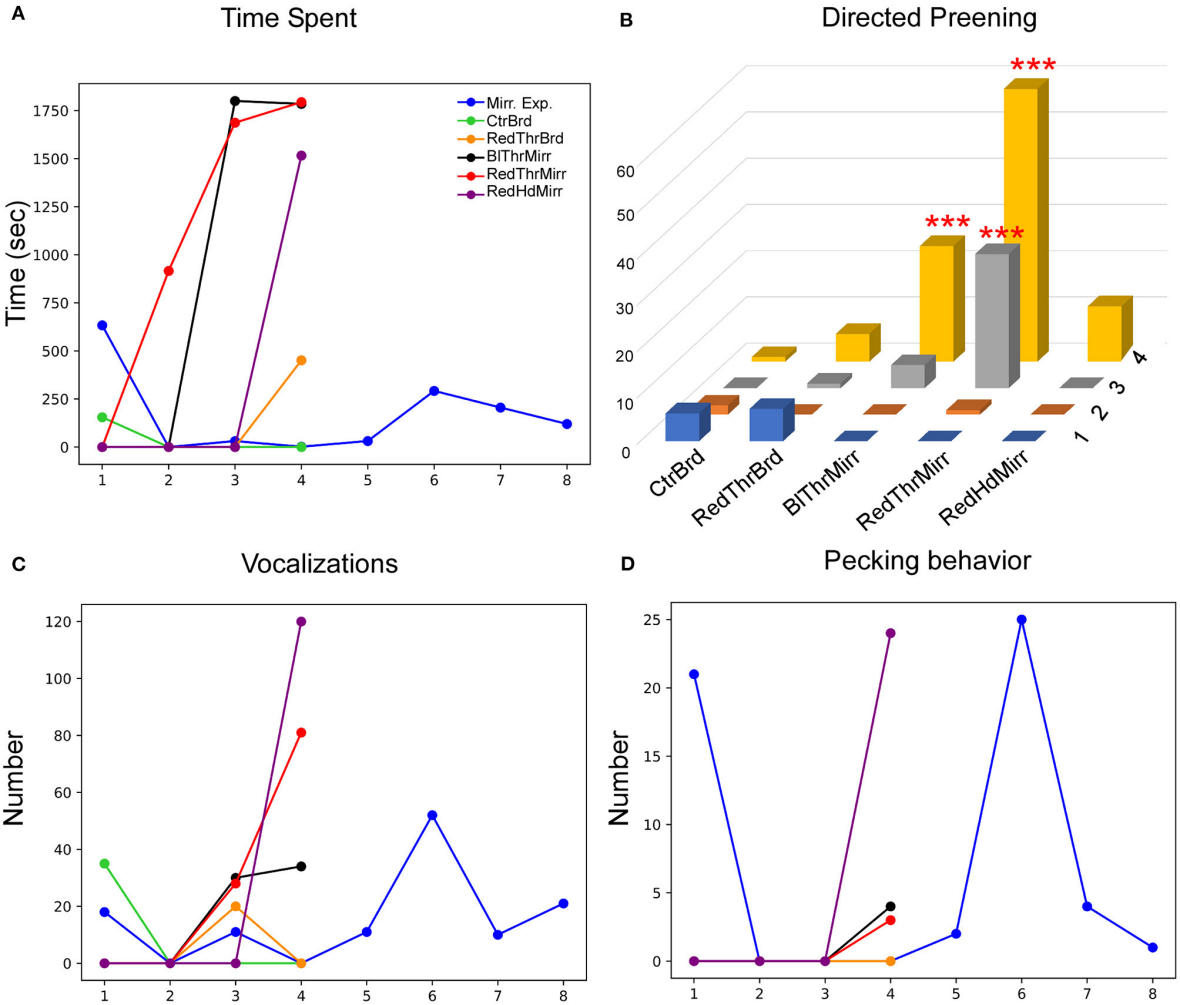
Behavioral responses of another zebra finch (G123) while testing for MSR. **(A)** Whereas this bird spent less time facing the mirror during the mirror exposure trials, there was an increase in this parameter during Trial 3 and Trial 4 for the mirror conditions (RedThrMirr and BlThrMirr). **(B)** Mark-directed preening increased significantly for the RedThrMirr condition (Trials 3 and 4) and the BlThrMirr condition (Trial 4) vs. controls (***; *P* < 0.001; Fisher's exact test). **(C)** Vocalization directed toward the mirror image was low in mirror exposure sessions and initial mirror trials but increased in the last mirror test trials. **(D)** There were no significant changes in pecking directed toward the mirror in any of the conditions tested.

Although G123 did not spend a lot of time examining its reflection in the mirror exposure trials, there was a non-significant increase in the amount of time it spent facing the mirror during most of the test trials {One Way RM ANOVA, [F_(5,27)_ = 2.439], *P* = 0.083; Figure 6A}. Similarly, whereas this bird displayed high levels of directed preening in the test phase, it mostly demonstrated undirected preening in the mirror exposure trials (undirected preening; Figure 3A; Table 1). Since this bird did not display any aggressive behavior toward its reflection and behaved passively in mirror exposure trials, we tested this bird further on the mark test. We found that G123 demonstrated high levels of preening (Fisher's exact test, *P* < 0.001, Supplementary Figure 1C) in the RedThrMirr condition compared with board and mirror exposure trials. Furthermore, we observed a significant increase in directed preening on the neck during the last two trials of the RedThrMirr condition (pairwise comparison using Fisher's exact test, *P* < 0.0001; Figure 6B) and the last trial of the BlThrMirr condition (pairwise comparison using Fisher's exact test, *P* < 0.0001).

Amongst other behaviors, the number of vocalizations directed by G123 toward the mirror image were high only in the last few mirror exposure trials as it did not face the mirror during most of the trials. Vocalization was very low in the initial test trials but increased in the last trials of all mirror test conditions (Figure 6C). It displayed very few conspecific-directed search responses, for example, turning clockwise or anticlockwise in front of the mirror and looking behind the mirror (see Table 1). Furthermore, it demonstrated increased contingency behavior in those mirror trials where it approached the mirror (Table 1), whereas in board trials, it did not approach the board. Finally, there was almost negligible pecking on the mirror and its frame (Figure 6D).

### House Crows

#### Mirror Exposure Phase

**Figure 7:**
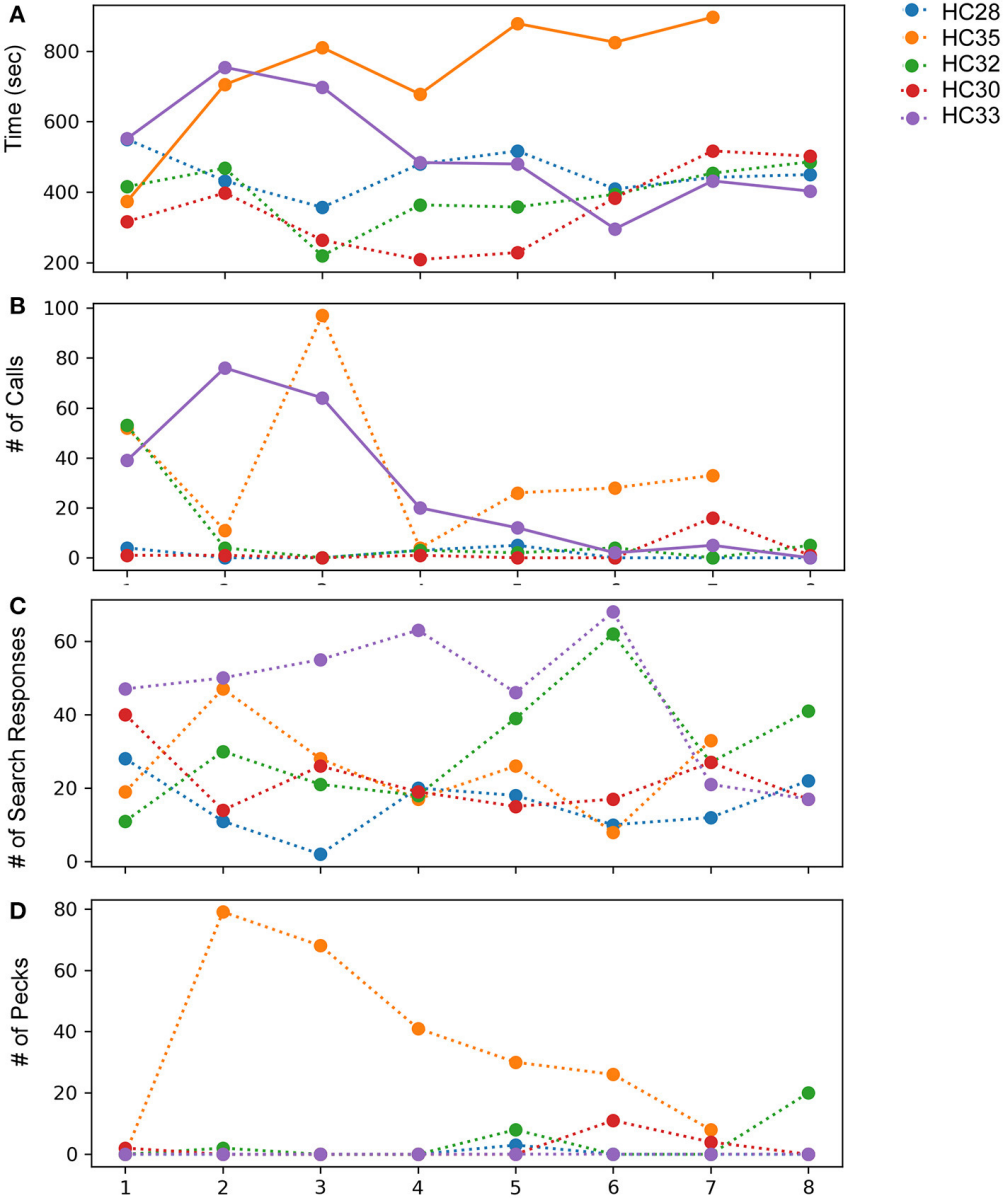
Effects of observing mirror images in adult house crows across eight successive sessions of the mirror exposure phase. **(A)** Time spent while facing the mirror, **(B)** number of calls produced while facing their reflections, **(C)** number of search responses (looking behind the mirror and turning clockwise or anticlockwise) while facing the mirror, and **(D)** number of pecks on the surface or sides of the mirror, indicating aggressive behavior. Colors indicate different crows used for the experiment, bold lines indicate significantly increasing or decreasing linear trends (*P* < 0.05; paired *t*-test) as trials progressed and dotted lines represent no significant differences for different behaviors analyzed.

All five crows tested displayed a keen interest in their mirror image measured by the time spent in front of the mirror during mirror exposure trials (Figure 7A). In one crow, there was a significant decrease in the time spent examining its reflection over the course of the mirror exposure trials (HC33, *t*-test, *t* = −2.708, *P* = 0.035). In contrast, the time spent facing the mirror increased significantly in the case of another crow (HC35; *t*-test, *t* = 3.013, *P* = 0.029; Figure 7A). For the remaining birds, there was no significant change in the time spent attending to their reflections over the course of the mirror exposure trials.

Unlike zebra finches, house crows rarely vocalized while facing their reflections in the mirror. Only two crows (HC33 and HC35; Figure 7B) vocalized toward their mirror image in more than three mirror exposure trials. Whereas there was a significant decrease in the number of vocalizations over the course of the mirror exposure trials in case of HC33 (*t*-test, *t* = −3.470, *P* = 0.01), there were no significant changes in the number of HC35's vocalizations directed toward the mirror. Amongst the other birds used for this experiment, other than in the first trial, HC32 directed very few vocalizations toward its reflection. For the last two birds, HC28 did not vocalize much during mirror exposure trials and HC30 did not vocalize at all while facing the mirror, except in the seventh trial.

The number of search responses, including looking behind the mirror and turning clockwise or anticlockwise was high for all five house crows used in these experiments (Figure 7C). However, these behaviors were very variable and did not follow an increasing or decreasing trend. Aggressive behavior demonstrated by house crows was measured by the number of times they pecked at their mirror image or the frame of the mirror. Only one crow (HC35) demonstrated aggressive behavior toward its reflection by pecking at it several times. However, this behavior decreased as mirror exposure trials progressed. All other crows used in these experiments pecked at their reflections occasionally during the trials (Figure 7D).

All house crows in our study demonstrated low levels of preening in the mirror exposure phase (Figure 3B) and we did not find any significant difference in directed vs. undirected preening on the neck, head, and wings. House crows also performed a larger number of head turns during the mirror exposure trials, as in the case of zebra finches used in our study. However, we did not observe any decrease in this behavior as the mirror exposure trials progressed in any of the crows (Figure 3D).

#### Comparison Between Mirror Exposure and Test Phases

##### Time Spent Examining the Mirror Image, Vocalization, and Contingency Testing

**Figure 8:**
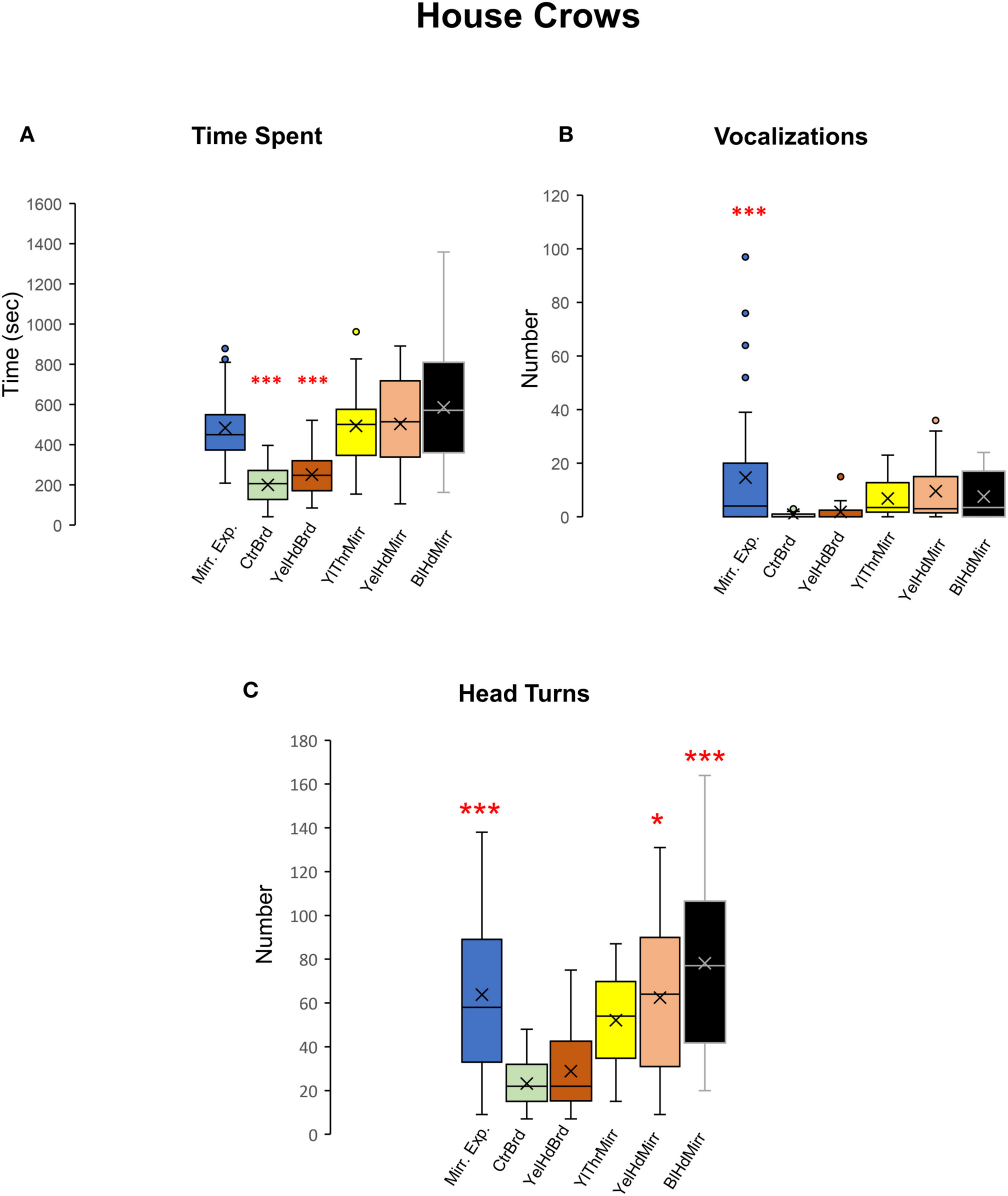
Time spent facing the mirror, vocalizations, and head turns directed toward mirror images in house crows. **(A)** House crows (*n* = 5) spent more time facing the mirror in the mirror exposure and mirror test conditions compared with that during the board test conditions. **(B)** They produced a significantly greater number of vocalizations while facing the mirror during the mirror exposure period compared with those during any of the test phase conditions (*Post-hoc* Tukey test ***, *P* < 0.001). **(C)** There was a significant increase in contingency behaviors (head turns) during the mirror exposure trials, YelHdMirr and BlHdMirr conditions compared to trials when house crows were facing a board. There was a slight but non-significant increase in head turns in the YelThMirr condition (*post-hoc* Tukey test *; *P* < 0.05; ***, *P* < 0.001).

All five crows tested displayed a keen interest in their mirror image measured by the time spent in front of the mirror during mirror exposure trials and test trials {One-way RM ANOVA, [F_(5,38)_ = 16.897], *P* < 0.001; Tukey's *post-hoc* test, *P* < 0.001; Figure 8A, Table 2) compared to board test trials. We were forced to end the mirror self-recognition experiments on one of the birds (HC33) after 12 test trials because it started pulling at the mirror or board and even caused it to fall. All experimental crows vocalized while facing their reflections in both test as well as mirror exposure trials. Our results demonstrated that there was an increase in the number of vocalizations in the mirror exposure trials compared with the test trials {One-way RM ANOVA, [F_(5,38)_ = 7.368], *P* < 0.001; Tukey's *post-hoc* test, *P* < 0.001; Figure 8B}. Birds also vocalized more during the mirror conditions compared to the board conditions, although this increase was not significant. Additionally, there was a significant increase in the number of head turns (contingency testing) in house crows when they faced their reflections {One-way RM ANOVA, [F_(5,38)_ = 13.111], *P* < 0.001; Tukey's *post-hoc* test; Figure 8C}. The number of head turns was significantly higher in the mirror exposure trials, BlHdMirr and YelHdMirr conditions compared to the board conditions (CtrBrd and YelHdBrd).

**Table 2:** Quantitation of different behaviors performed by Indian house crows during mirror self-recognition.

**Behavior**	**Bird**	**Mirror exposure**	**CtrBrd**	**YelHdBrd**	**YelHdMirr**	**YlThrMirr**	**BlHdMirr**
Time spent (sec)	HC28	3637	899	962	1099	1340	1401
	HC30	2820	818	1040	1848	1684	2509
	HC32	3160	601	442 (3)^**^	1932 (3)^**^	1725 (3)^**^	1088 (2)^**^
	HC33	4099	604 (3)^*^	556 (2)^*^	1384 (2)^*^	914 (3)^*^	737 (2)^*^
	HC35	5166 (7)^**^	891	1518	2628	2902	3648
Calls	HC28	12	0	1	4	3	2
	HC30	20	7	62	61	67	70
	HC32	71	3	0 (3)^**^	16 (3)^**^	33 (3)^**^	7 (2)^**^
	HC33	218	5 (3)^*^	0 (2)^*^	18 (2)^*^	15 (3)^*^	2 (2)^*^
	HC35	251	1 (7)^**^	8	24	44	40
Head turns	HC28	381	88	76	148	108	169
	HC30	280	87	76	200	195	294
	HC32	626	107	82	311	210	256
	HC33	372	66 (3)^*^	30 (3)^**^	75 (3)^**^	140 (3)^**^	73 (2)^**^
	HC35	829 (7)^**^	92	223 (2)^*^	328 (2)^*^	285 (3)^*^	458 (2)^*^
Search responses	HC28	123	101	95	63	121	98
	HC30	175	87	77	107	137	121
	HC32	249	74	72 (3)^**^	92 (3)^**^	107 (3)^**^	67 (2)^**^
	HC33	367	55 (3)^*^	33 (2)^*^	84 (2)^*^	31 (3)^*^	44 (2)^*^
	HC35	178 (7)^**^	65	50	101	63	85
Exploratory/aggressive behavior	HC28	3	12	7	0	0	0
	HC30	17	0	48	11	15	104
	HC32	30	0	0 (3)^**^	12 (3)^**^	50 (3)^**^	64 (2)^**^
	HC33	0	0 (3)^*^	25 (2)^*^	0 (2)^*^	0 (3)^*^	0 (2)^*^
	HC35	252 (7)^**^	156	122	68	29	106
Scratching (Directed)	HC28	0	0	1	0	0	1
	HC30	1	1	0	0	2	0
	HC32	0	0	0 (3)^**^	0 (3)^**^	1 (3)^**^	0 (2)^**^
	HC33	2	0 (3)^*^	0 (2)^*^	0 (2)^*^	0 (3)^*^	0 (2)^*^
	HC35	0 (7)^**^	0	0	0	0	0
Scratching (Undirected)	HC28	1	0	0	0	0	0
	HC30	2	1	0	0	2	0
	HC32	2	0	0 (3)^**^	1 (3)^**^	0 (3)^**^	0 (2)^**^
	HC33	0	0 (3)^*^	0 (2)^*^	0 (2)^*^	0 (3)^*^	0 (2)^*^
	HC35	0 (7)^**^	0	0	0	0	0

* *For HC33, the experiment had to be stopped since it started pulling at the mirror or board, causing them to fall. The number within the parenthesis indicated the number of trials performed for each condition available for analysis*.

** *Parentheses indicate the number of trials available for analysis in cases where video recordings stopped after 2–5 min*.

##### Search Responses and Aggressive Behavior

The number of search responses, including looking behind the mirror and turning clockwise or anticlockwise was high for all five house crows used in these experiments (Table 2). However, these behaviors were very variable and did not follow an increasing or decreasing trend. Aggressive behavior demonstrated by house crows was measured by the number of times they pecked at their mirror image or the frame of the mirror. Only one crow (HC35) demonstrated aggressive behavior toward its reflection (Table 2).

#### Mark Responses in House Crows

Whenever the mark was placed on the wings or bellies which could be directly observed by experimental house crows, they were immediately removed, indicating that house crows recognized the stickers as foreign objects placed on their bodies. None of the house crows used in our experiments appeared to recognize the mark placed on their heads or necks by preening themselves while facing the mirror. However, overall levels of preening in house crows were also very low, compared to those in zebra finches (Table 2). Although one crow (HC30) demonstrated directed preening on the neck or scratched itself, this was not mark-specific (Supplementary Figures 1D,E; Supplementary Video 4; Table 2). The video demonstrates that it scratched itself once close to the sticker (Supplementary Video 5) but was not facing the mirror. This may have been part of general grooming since it also scratched itself on its head (Supplementary Video 6; YelThrMirr) and near its beak during mirror exposure and CtrBrd trials and preened its neck during the YelHdMirr condition (Supplementary Video 7).

## Discussion

### Mirror Image Stimulation and Mirror-Directed Behaviors

Apart from increased mark-directed preening (discussed below), increases in mirror directed self-exploration (Povinelli et al., 1993), contingency behaviors (Ari and D'Agostino, 2016), concomitant decreases in aggressive behavior, conspecific search responses (Gallup, 1970) and vocalizations are also considered to be positive indicators of visual self-recognition. In our study, we found significant decreases in the time spent viewing the reflection in one zebra finch (Y45) and one crow (HC33), suggesting that they adapted to their reflections, which is also observed in other species of birds (Prior et al., 2008; Medina et al., 2011; Vanhooland et al., 2019). There were also clear decreases in social responses (vocalization) in two zebra finches (Y45 and Y48) and one crow (HC33). Moreover, contingency behavior such as head turns, considered to indicate matching visual cues and kinesthetic input between the reflection and oneself, was higher in mirror conditions compared to controls in the majority of zebra finches but there was no clear decrease in this behavior during the course of the mirror exposure trials. Examples of aggressive or exploratory behavior measured by pecking on the mirror and frame were generally low in finches. We found that house crows also displayed a greater number of contingency behaviors in mirror trials compared to board trials. Additionally, search responses decreased in number over the course of the experiments in HC33 (Table 2). Although these findings may be interpreted as the initial stages of mirror self-recognition (Buniyaadi et al., 2019), it is also possible that the house crows may simply have adapted to the image of a conspecific in the mirror.

Rochat has suggested that animals may treat reflections in mirrors as extensions of their environment (Rochat, 2003). Initial responses to mirror images are generally aggressive such as bobbing, vocalizing, and threatening in chimpanzees (Gallup, 1970), jumping toward the mirror [Eurasian magpies; (Prior et al., 2008)], pecking and wing flapping [jungle crows; (Kusayama et al., 2000)], and fighting their reflection [African cichlid fish; (Desjardins and Fernald, 2010)]. Earlier studies have shown that these responses decrease over time and are replaced by self-exploratory behaviors such as grooming, making faces, and picking extraneous material from the eyes, nose, and teeth in chimpanzees (Gallup, 1970) or self-directed preening in birds (Prior et al., 2008) as animals learn to associate themselves with their reflections. However, we did not find that birds progressed from predominantly social to predominantly self-exploratory behaviors in our experiments, with the progression of the mirror exposure trials. Neither did we observe significant decreases in vocalizations, search responses, exploratory/aggressive responses or an increase in self-directed preening during the progression of mirror exposure trials in the majority of experimental birds, such as those observed in apes, elephants and dolphins (Reiss and Morrison, 2017). Furthermore, other than directed preening (a general grooming behavior), neither zebra finches nor house crows performed behaviors which were unique to the mirror conditions.

### Inter-species and Inter-individual Variability Within the Same Species in the Performance on the Mark Test

Many authors have doubted the reliability of the mark test as an indicator of mirror self-awareness. For example, it was found that very few 18–72 month-old children from rural areas of non-western countries (two out of 82 in Kenya) passed the test or displayed any orientation toward the mark as opposed to western children of similar ages (Broesch et al., 2011). None of these children had psychological problems, suggesting a role for cultural variation and prior exposure in the performance on the mark test. Similarly, high degrees of inter-individual variability exist amongst various species of animals and birds in mirror self-recognition. For example, only 21 of 92 chimpanzees tested displayed self-exploration when faced with their reflections, of which only nine passed the mark test (Povinelli et al., 1993). Amongst Asian elephants, only one out of three passed the mark test (Plotnik et al., 2006) and amongst Eurasian magpies, only two of five birds passed the mark test (Prior et al., 2008). When a larger set of eight magpies were tested using a similar paradigm by another group, none of them passed the mark test (Soler et al., 2020). For many species of birds [keas, carrion crows, magpies, great tits, and jackdaws; (Soler et al., 2014, 2020; Kraft et al., 2017; Buuren et al., 2018; Brecht et al., 2020)] and mammals [monkeys, gorillas, bonobos, and most chimpanzees; (Suarez and Gallup, 1981; Povinelli et al., 1993; Anderson and Gallup, 2015)], the reasons underlying their failure to display mark-directed behavior ranges from gaze aversion, lack of interest in the mirror image or the mark since it does not pose a threat, to lack of motivation (Schilhab, 2004; Anderson and Gallup, 2015; Chang et al., 2015; Brecht et al., 2020). This suggests that failing the mark test does not automatically suggest that the subject is not self-aware. Given the high degree of variability in MSR even amongst those species which are known to pass the mark test, our results on zebra finches and house crows (as discussed below) are not surprising.

We found a significant increase in mark-directed preening in two of the six zebra finches during test conditions compared to control conditions (board and tactile stimulation with a wet brush). The first bird (Y45) demonstrated negligible preening in the mirror exposure trials and there was a progression toward increased preening in the mark test trials (RedThrMirr and BlThrMirr). The second zebra finch (G123) mostly demonstrated undirected preening in the mirror exposure trials and increased levels of directed preening on the neck in the last trials of the mark test conditions. Besides displaying mark-directed behavior, one of the two birds (Y45) demonstrated a clear decrease in attention toward its reflection, vocalization and non-significant decreases in contingency and aggressive behaviors during the mirror exposure phase.

The lack of spontaneous mark-directed preening and an increase in mark-directed behavior with an increase in the number of test trials in Y45 and G123 suggests that they may have learnt the position of the mark by actually perceiving that the reflected image was their own. Alternatively, they may have learnt the position of the mark without the aid of a mirror and observing their reflections may have somehow elicited an increase in preening near the mark. A third possibility is that mark-directed preening was performed by chance and the increase in self-exploratory behavior may have occurred as a result of increasing the number of mirror trials in these birds. Therefore, the self-directed and mark-directed behaviors observed in this study are not conclusive evidence of MSR in zebra finches.

Although we had used black paint as a control for color, greater levels of mark-directed preening were observed in these birds when a black spot was painted on black feathers on their necks. Furthermore, in a finch with black and white patches, there was an increase in preening directed toward a white spot painted on white feathers under its beak. Birds including zebra finches are known to have acute color vision and possess receptors for the ultraviolet spectrum, enabling them to see greater detail and contrast than do humans. However, corvids can only sense light in the visible spectrum (Ödeen et al., 2011; Kelber, 2019). It is therefore possible that heightened visual acuity in finches resulted in their being able to recognize the black and white marks on black and white feathers, respectively, in mirror images, even though we could not discriminate between these colors painted on the finches.

Despite the fact that our findings suggest that a small proportion of zebra finches (two out of six) demonstrate an increase in preening near the mark when faced with their reflections, studies on magpies (Prior et al., 2008; Soler et al., 2020) have demonstrated that it is fraught with issues related to false-positives and false-negatives. Since the sample size in the present study is small, it is important to repeat the experiments with a larger number of birds to ensure that these results are not just “by chance” as discussed in detail in Soler et al. (2020). Another caveat is that across all experimental zebra finches in our study including the two which demonstrated increased mark-directed preening in the RedThrMirr condition, there was no significant increase in self-directed preening in the RedHdMirr condition. It is, of course, possible that these birds were either unaware of the mark on their heads or were somehow less motivated to preen or scratch the area. Besides increasing the sample size, differentiating self-directed preening specifically performed in front of mirrors from that important for general grooming would be also important for future studies on MSR in birds. This would involve a rigorous screening of all self-directed behaviors during the mirror exposure trials before performing the mark-test.

In case of house crows, we found that these birds did not preen themselves frequently, whether during mirror exposure or test trials. As mentioned in **Methods**, Soler et al. (2014) reported that jackdaws recognized stickers placed on their neck as a result of tactile stimulation, in the mirror as well as control (board) conditions. In our study, house crows removed the stickers if they were able to directly observe them on their wings or on their bellies. However, none of these birds passed the mark test when the sticker could only be observed in their reflections and neither did they attempt to remove the stickers in the board conditions. These findings may have resulted from the low levels of self-exploration in these birds. These findings are also supported by the fact that house crows spent less time examining their reflections (~27%). Whereas, our findings in house crows are comparable to those in jungle crows (Kusayama, Bischof, and Watanabe 2000), New Caledonian crows (Medina et al., 2011), carrion crows (Vanhooland et al., 2019; Brecht et al., 2020), and jackdaws (Soler et al., 2014), which did not display mirror directed self-exploration, a recent study (Buniyaadi et al., 2019) has demonstrated that house crows pass the mark test. However, there were a number of differences between their paradigm and ours. They had used shorter periods for familiarization with the mirror and for the mark test, compared to our study. Surprisingly, even though the sticker itself was lighter and smaller in the other study [(Buniyaadi et al., 2019); 4.5 mg and 7 mm in diameter] and we had used heavier stickers (12–13 mg) of larger size (9 mm in diameter), the house crows in our study did not attempt to remove the stickers after observing their reflections. Although Buniyaadi et al. (2019) claim that four out of six house crows pass the mark test, only two of these four birds display high mark-directed behavior (without removing the stickers) whereas this was negligible in two others (crows 1 and 4; once or twice in two sessions). Even amongst the other birds used for these experiments, one demonstrated extraordinarily high levels of preening (a total of 82 times during the trials) over its entire body during mirror exposure [crow 6, (Buniyaadi et al., 2019)]. These results also suggest that some of the mark-directed preening reported for this house crow may have been a part of generalized preening to clean its plumage. Vanhooland et al. (2019) have also reported high contingency behaviors (peekaboos) but no mark-directed behavior in carrion crows. Taken together with our results, these findings suggest that the majority of house crows appear not to recognize themselves when subjected to the standard mark test (cf. Prior et al., 2008).

### Conclusions on Self-Recognition in Zebra Finches and House Crows

We have found that a small number of zebra finches appeared to demonstrate mark-directed preening behavior. Taken together with results from MSR experiments on magpies (Soler et al., 2014) and great tits (Kraft et al., 2017), we cannot completely rule out the possibility that zebra finches which passed the mark test did so by chance, since self-preening is performed very frequently in these birds as a general grooming behavior. Thus, more rigorous testing during the mirror exposure phase with a larger sample size is required to determine whether zebra finches possess the third level (identification) of self-awareness, that is, recognizing marks/stickers placed on one's body which can only be observed in reflections described by Rochat (2003). House crows do not pass the mark test using our experimental paradigm, and thus it is possible that these birds do not recognize themselves in mirrors or adapt to their reflections. These results suggest that house crows appear to demonstrate levels 1 and 2 (differentiation and situation, respectively; Rochat, 2003) of self-awareness when faced with their reflections. Our results also emphasize the large degree of variability on the performance of mirror-image associated behaviors including the mark test. Furthermore, results of the mark test must be interpreted cautiously, by taking various mirror-directed behaviors into consideration and also repeated with a large sample size (de Waal, 2019).

## Data Availability Statement

Seven videos have been provided as Supplementary Data. Primary data will be provided on request and is also available in the Dryad Repository (doi: 10.5061/dryad.xwdbrv1bx).

## Ethics Statement

The animal study was reviewed and approved by Institutional Animal Ethics Committee, NBRC, Manesar [in accordance with guidelines laid down by the Committee for the Purpose of Control and Supervision of Experiments on Animals (CPCSEA), India].

## Author Contributions

PP and SI designed the experiments, interpreted the data, and drafted the manuscript. PP performed the behavioral experiments and the statistics. PP and AM analyzed the behavioral data. SI conceived of and coordinated the study. All authors contributed to the scientific discussions and gave approval for the final manuscript and are accountable for the work performed therein.

## Conflict of Interest

The authors declare that the research was conducted in the absence of any commercial or financial relationships that could be construed as a potential conflict of interest.

## Supplementary Material

Data Sheet 1 Captions for the supplementary materials.
