# Supplementary material for: Investigating Behavioral Responses to Mirrors and the Mark Test in Adult Male Zebra Finches and House Crows
Source: Front Psychol. 2021 Apr 15;12:637850. doi: 10.3389/fpsyg.2021.637850 (PMC8082158; doi:10.3389/fpsyg.2021.637850)
Supplement: Data Sheet 1 — Captions for the supplementary materials. [file Data_Sheet_1.docx]

**Supplementary Figure 1. (A**) Schematic of head-turning behavior. When experimental birds rotate their head almost 90 degrees towards the right or left and turns back or from left or right to the front and back, it is counted as one head turn. **(B)** and **(C)** A comparison of percentage preening in Y45 and G123 across various conditions. **(D)** Directed and (**E**) undirected preening on the neck by house crows. Only one crow (HC30) preened itself on the neck during the test conditions YelHdMirr and YelThrMirr, but these were not specifically directed towards the mark.

**Video 1**. Vocalizations produced by a zebra finch facing the mirror (Y42).

**Video 2**. Head turns performed by a zebra finch while facing the mirror (Y45).

**Video 3**. Directed preening on a mark placed on the neck by a zebra finch (Y45).

**Video 4**. Preening not directed to the mark in the YelThrMirr condition (HC30).

**Video 5**. Scratching close to the mark not facing the mirror (undirected, HC30).

**Video 6.**  Scratching on the head in the YelThrMirr condition (HC30).

**Video 7**. Directed preening on the neck in the YelHdMirr condition (HC30).
