# Supplementary figures and images for: Investigating Behavioral Responses to Mirrors and the Mark Test in Adult Male Zebra Finches and House Crows
Source: Front Psychol. 2021 Apr 15;12:637850. doi: 10.3389/fpsyg.2021.637850 (PMC8082158; doi:10.3389/fpsyg.2021.637850)

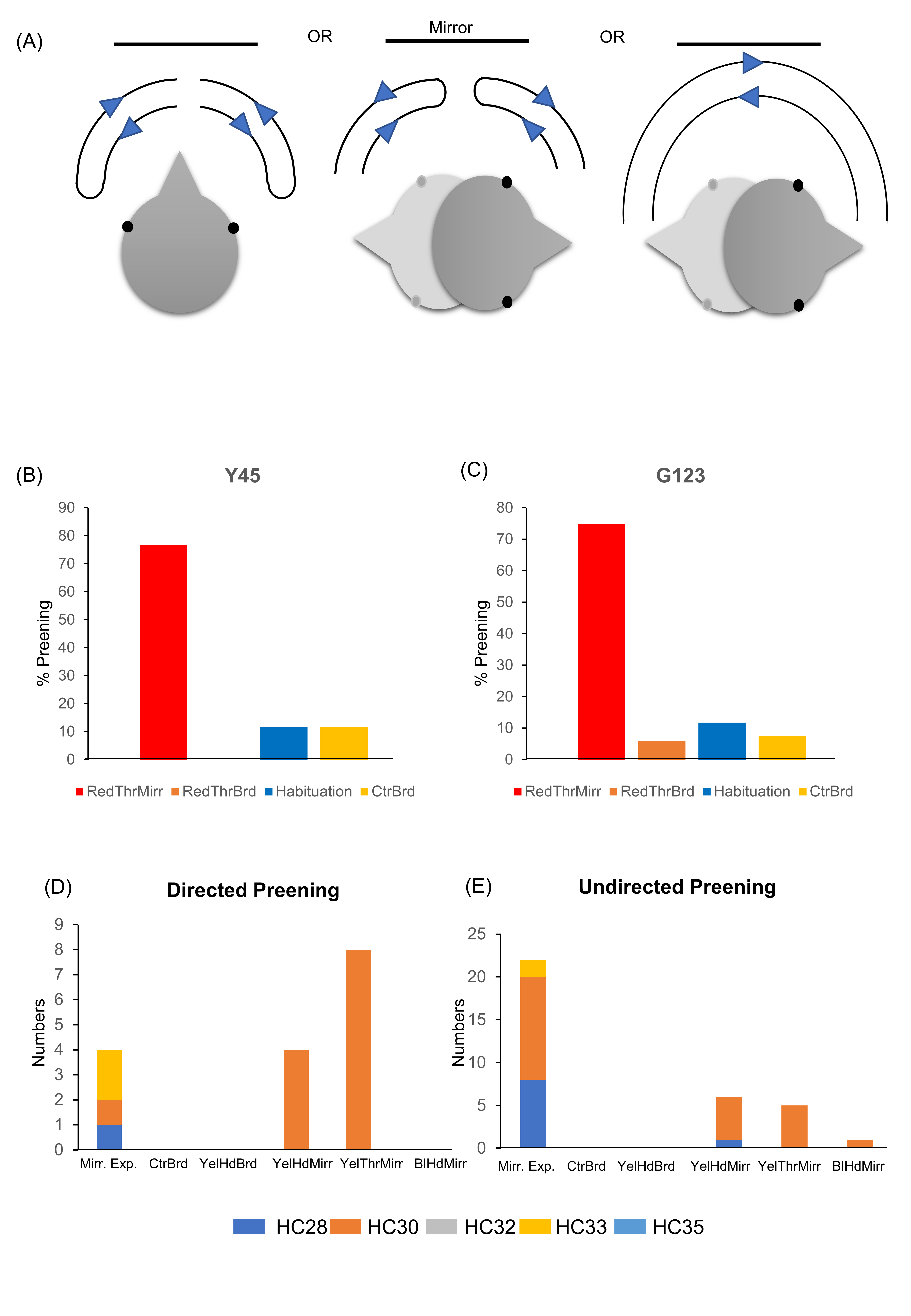

Supplement: Supplementary file 9 [file Image_1.TIF]
